# Physicochemical and Biological Study of ^99m^Tc and ^68^Ga Radiolabelled Ciprofloxacin and Evaluation of [^99m^Tc]Tc-CIP as Potential Diagnostic Radiopharmaceutical for Diabetic Foot Syndrome Imaging

**DOI:** 10.3390/tomography7040070

**Published:** 2021-12-01

**Authors:** Przemysław Koźmiński, Weronika Gawęda, Magdalena Rzewuska, Agata Kopatys, Szymon Kujda, Marta K. Dudek, Paweł Krzysztof Halik, Leszek Królicki, Ewa Gniazdowska

**Affiliations:** 1 Centre of Radiochemistry and Nuclear Chemistry, Institute of Nuclear Chemistry and Technology, Dorodna 16, 03-195 Warsaw, Poland; w.maliszewska@ichtj.waw.pl (W.G.); p.halik@ichtj.waw.pl (P.K.H.); e.gniazdowska@ichtj.waw.pl (E.G.); 2 Institute of Veterinary Medicine, Department of Preclinical Sciences, Division of Microbiology, Warsaw University of Life Sciences—SGGW, Ciszewskiego 8, 02-786 Warsaw, Poland; magdalena_rzewuska@sggw.edu.pl; 3 Department of Nuclear Medicine, Medical University of Warsaw, Banacha 1a, 02-097 Warsaw, Poland; agata.kopatys@uckwum.pl (A.K.); szymon.kujdaa@gmail.com (S.K.); leszek.krolicki@wum.edu.pl (L.K.); 4 Centre of Molecular and Macromolecular Studies, Polish Academy of Sciences, Sienkiewicza 112, 90-363 Lodz, Poland; mdudek@cbmm.lodz.pl

**Keywords:** ciprofloxacin, technetium-99m, gallium-68, radiopharmaceutical, bacterial infection, diabetic foot

## Abstract

This paper presents the application of ciprofloxacin as a biologically active molecule (vector) for delivering diagnostic radiopharmaceuticals to the sites of bacterial infection. Ciprofloxacin-based radioconjugates containing technetium-99m or gallium-68 radionuclides were synthesised, and their physicochemical (stability, lipophilicity) and biological (binding study to *Staphylococcus aureus* and *Pseudomonas aeruginosa*) properties were investigated. Both the tested radiopreparations met the requirements for radiopharmaceuticals, and technetium-99m-labelled ciprofloxacin turned out to be a good radiotracer for the tomography of diabetic foot syndrome using SPECT.

## 1. Introduction

Bacterial infections usually induce a response such as a fever or inflammation from the body. In general, these infections are treated with antibiotics and sometimes with other bactericidal agents. The diagnosis of bacterial infection is often a long process, and it is also usually difficult to identify the specific area that is infected [1].

There is therefore an urgent need to develop new and efficient diagnostic methods, targeting inflammation and infection sites. Single-photon emission computed tomography (SPECT) and positron emission tomography (PET) imaging methods stand out due to their resolution and the possibility of the precise localisation of any lesions, provided they are targeted with high specificity. There are already a large number of PET and SPECT radiopharmaceuticals that have been tested as bacterial infection diagnostics [2,3,4,5]. They have been assessed in terms of their physicochemical and biological properties (stability, efficiency, and specificity of binding—the ratio of binding to infected compared to uninfected tissue in cases of inflammation) and the possibility of diagnosing aseptic and infectious inflammation, as well as inflammatory processes accompanying other diseases. However, the ideal radiopharmaceutical for functional imaging of inflammation and/or infection has still not been found, and taking into account the requirements for personalised medicine, it is necessary to constantly search for new radiopreparations.

An example of a disease where rapid and correct diagnosis is crucial is diabetic foot syndrome (DFS). Diabetes is a disease that is considered a global epidemic and a high priority issue for the 21st century. According to World Health Organisation (WHO) forecasts and other studies [6], the number of people worldwide with diabetes reached 422 million in 2014, and it is predicted that 578 million people will have the condition by 2030. Poorly controlled diabetes usually leads to the development of numerous complications and side effects, one of the most serious being DFS [7]. DFS is associated with ulcer formation, and in diabetic patients the lifetime risk of developing an ulcer is about 12–25% [8]. The increased level of glucose in the blood in diabetics (hyperglycaemia) causes blood clots that promote blood vessel damage and can lead to atherosclerosis and ulceration. This process results in ischemia of the tissues, which leads to their malfunction and hypoxia. Long-term hypoxia is associated with neuropathy and tissue necrosis, which makes the tissue prone to infection. As tissue hypoxia and necrosis usually develop asymptomatically, unfortunately they may go undetected for a long period of time. When small areas are affected, treatment with antibiotics and minor surgical interventions are used. In severe cases of necrosis and extensive bacterial infection, depending on the extent of the infection, limb fragments or even the entire foot may be amputated. In developed countries, diabetic foot syndrome is the most common cause of non-traumatic limb loss. In such cases, rapid and correct diagnosis and treatment of the area affected by infection is very important.

In cases of DFS, various diagnostic methods and treatments are applied, including radiopharmaceuticals [9,10,11]. Unfortunately, the radiopharmaceuticals developed so far have various drawbacks that limit their clinical application. Radioisotope-labelled leukocytes are considered the ‘gold standard’ for visualising most infectious and inflammatory lesions, but this method is laborious and takes about three hours. In addition, collecting white blood cells from a patient and labelling them in vitro requires specialised equipment and can be dangerous due to contact with potentially contaminated blood. Antibiotic-based radiopharmaceuticals have been tested for their accumulation at target and non-target sites (T/NT ratio) and their ability to distinguish infection from sterile inflammation, but the results were inconclusive and dependent on various parameters, including the type and site of infection. For these reasons, the search for new radiopharmaceuticals is still important. The selected radiopharmaceutical should reach a specific area and thus be able to accurately locate the infected regions, allowing for the implementation of appropriate treatment and an accurate assessment of the need for amputation. As amputation of a part of a limb significantly affects the patient’s quality of life and leads to disability, the assessment of the extent of the lesions is extremely important.

One group of antimicrobials used to treat infectious diseases are quinolones. They are derivatives of quinoline just like quinine, a substance known as the first effective treatment for malaria. There are four generations of quinolones [12,13], most of which are fluoroquinolones (2nd generation). They have a bactericidal effect and are therefore used to treat bacterial infections. The structure of quinolones allows various modifications to the molecule to be introduced without it losing its properties and therapeutic effect [13], so they are applied in radiopharmaceuticals for imaging bacterial infections after labelling with ^99m^Tc [11,14,15,16,17,18] or ^18^F [10,19,20].

**Figure 1:**
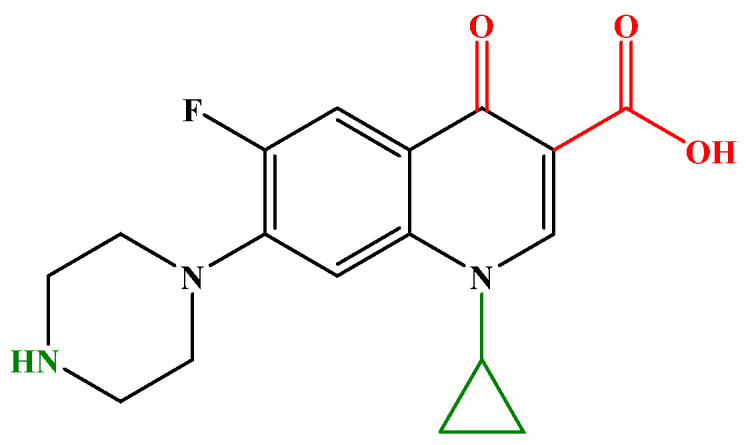
Ciprofloxacin structure with highlighted in red essential fragment for enzyme binding and highlighted in green possible modification positions.

**Figure 2:**
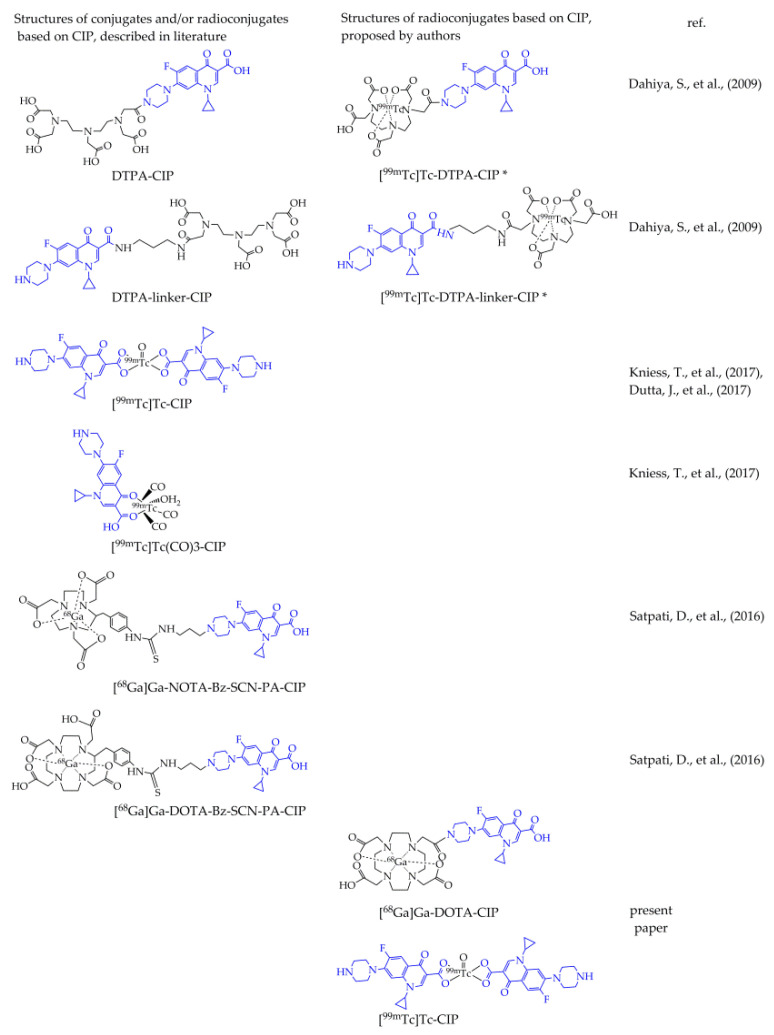
Structures of various CIP conjugates and radioconjugates described in the literature and this paper. The structure of the CIP molecule is marked with a blue color. * structures of [^99m^Tc]Tc-DTPA-CIP and [^99m^Tc]Tc-DTPA-linker-CIP radioconjugates were proposed by the authors of this paper, based on the structures of DTPA-CIP and DTPA-linker-CIP conjugates presented in the cited article.

In this study, the application of the commonly used antibiotic ciprofloxacin (CIP, Figure 1) labelled with the diagnostic radionuclide technetium-99m for DFS diagnosis with SPECT is presented. In general, safety considerations result in radiopharmaceuticals being used in very low concentrations (10^−6^–10^−7^ M). Radiopharmaceuticals administered into the body in such amounts do not cause any pharmacological effects. CIP is a 2nd generation fluoroquinolone that is used in the treatment of many bacterial infections, e.g., urinary tract infections, skin infections and infections of certain organs, including infections caused by *Pseudomonas aeruginosa*, *Staphylococcus aureus,* or *Escherichia coli*. Additionally, we also present in this report the synthesis and study of the physicochemical and biological properties of CIP labelled with the diagnostic radionuclide galium-68—a radiopreparation suitable for DFS diagnosis with PET, a technique characterised by a higher sensitivity, spatial resolution, and temporal resolution (a few seconds to minutes). There is only one article on CIP labelled with Ga-68 in the literature [21], where the authors used either a macrocyclic DOTA (p-SCN-Bz-DOTA) or NOTA (p-SCN-Bz-NOTA) chelator, additionally attached to CIP via a propylamine (PA) linker. In our study, based on our own experience [22] and in order to avoid introducing into the radiopreparation molecule additional chemical fragments containing thiourea bonds [23], we used a DOTA-NHS chelator. The role of CIP in these radiopreparations is to act as a biologically active molecule (vector), which targets the radiopharmaceutical directly to the infected area with high specificity, allowing its precise visualisation by SPECT or PET. The structures of the various CIP radioconjugates described in the literature are shown in Figure 2.

## 2. Materials and Methods

CIP was a gift from the Public Central Teaching Hospital of Medical University of Warsaw, macrocyclic chelator 2,2′,2″-(10-(2-((2,5-dioxopyrrolidin-1-yl)oxy)-2-oxoethyl)-1,4,7,10-tetraazacyclododecane-1,4,7-triyl)triacetic acid (DOTA-NHS) was purchased from CheMatech (Dijon, France), other chemicals and solvents were purchased from Merck (Darmstadt, Germany) and used as obtained. ^99m^Tc (emitter γ, t_1/2_ = 6 h, E_γ_ = 140 keV, in the form of [^99m^Tc]-pertechnetate in 0.9% NaCl solution) and ^68^Ga (emitter β^+^, t_1/2_ = 67.7 min, E_β__max_ = 1.9 MeV, in the form of [^68^Ga]GaCl_3_ in 0.1 M HCl solutions) radionuclides were eluted from the commercially available ^99^Mo/^99m^Tc (Institute of Atomic Energy, Radioisotope Centre POLATOM, Świerk-Otwock, Poland) and ^68^Ge/^68^Ga (Eckert & Ziegler, Berlin, Germany) generators, respectively. Deionized water was prepared in a Hydrolab water purification system (Hydrolab, Straszyn, Poland). Human serum was a gift from the Regional Centre for Blood Donation and Blood Treatment in Warsaw, Poland.

HPLC analyses, separations, and purifications of synthesized compounds were carried out using a VWR-Hitachi LaChrom Elite HPLC system, which consisted of a pump L2130, column thermostat L-2350, UV diode array detector (DAD) L-2455 and the EZChrom Elite data system. The radioactivity was monitored using a 3 × 3″ NaI(Tl) scintillation detector Raytest Gabi Star (Straubenhardt, Germany). The separation of the obtained products was accomplished on a LiChrospher^®^ 100 RP-18 analytical column (5 µm particle size, 4.6 mm × 250 mm) from Merck (Germany). The solvent and gradient conditions were as follows: solvent A, 0.1% (*v*/*v*) trifluoroacetic acid (TFA) in water; solvent B, 0.1% (*v*/*v*) TFA in acetonitrile. HPLC analyses were performed using gradient elution method as follow: 5% to 50% B in 0–10 min, 50% to 100% B in 10–13 min, 100% B in 13–17 min, 1 mL/min; UV detection (220–400 nm)—system 1 or γ-detection—system 2.

Instant Thin-Layer Chromatography (iTLC) applied for radiochemical purification (RPC) of the synthesized [^99m^Tc]Tc-CIP and [^68^Ga]Ga-DOTA-CIP radioconjugates was performed using glass microfiber chromatography paper impregnated with a silica gel (Agilent, Santa Clara, CA, USA) and adequate mobile phase. After completing the development, the iTLC strips were measured using the Storage Phosphor System Cyclone Plus (Perkin-Elmer Life and Analytical Sciences, Downers Grove, IL, USA).

^1^H NMR spectra were registered with a Bruker Avance III spectrometer, operating at 500.13 MHz of resonating frequency for ^1^H. The spectra were registered in D_2_O in 3- or 5-mm NMR tubes, and the ^1^H signals were calibrated on the residual solvent signal (4.80 ppm). During the measurements, the temperature was stabilized at 295 K by a BCU unit, controlled by a BVT3200 variable temperature unit.

### 2.1. Syntheses

#### 2.1.1. Preparation of DOTA-CIP

**Scheme 1:**
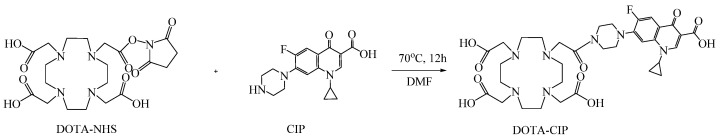
Coupling reaction of DOTA-NHS with CIP.

The molar excess of DOTA-NHS 3.1 mg (4.07 µmol) dissolved in 50 µL of DMF was placed in a round bottom flask, next 1 mg (3.02 µmol) CIP in 25 µL DMF and 2.24 µL (16 µmol) Et_3_N were added (Scheme 1). The reaction mixture was stirred at 70 °C overnight. After that, the solvent was removed under vacuum and the crude product was dissolved in acetonitrile/water, purified by HPLC and lyophilized. The pale-yellow powder was obtained and identified by MS analysis as DOTA-CIP.

#### 2.1.2. Preparation of [^68^Ga]Ga-DOTA-CIP Radioconjugate

**Scheme 2:**
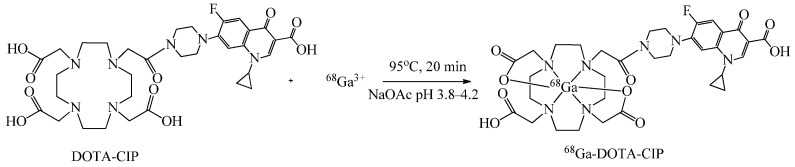
Scheme of radiolabelling reaction of DOTA-CIP.

The ^68^Ga radiolabelling of DOTA-CIP conjugate (Scheme 2) was performed according to the following procedure: 750 µL of [^68^Ga]GaCl_3_ (104.4 MBq) in 0.1 M HCl from the ^68^Ge/^68^Ga generator was added into the solution of 150 µg (~0.20 µmol) of DOTA-CIP conjugate previously dissolved in 750 µL of a 0.2 M acetate buffer (pH = 5) and heated for 20 min at 95 °C. The radiochemical yield and purity were determined by HPLC and ITLC methods.

To verify the identity of the [^68^Ga]Ga-DOTA-CIP radioconjugate, the analogue with stable gallium isotope under the same reaction conditions was synthesized and analyzed by HPLC (system 1 with UV detection), ^1^H-NMR and MS methods.

#### 2.1.3. Preparation of [^99m^Tc]Tc-CIP Radioconjugate

The [^99m^Tc]Tc-CIP radioconjugate was prepared using lyophilized kits. Each kit contains 2 mg (~6.04 µmol) of CIP and 0.08 mg of SnCl_2_. The radiolabeling was achieved as follow: 1 mL of a [^99m^Tc]TcO_4_^−^ fresh eluate with activity of 400–750 MBq was added to the kit vial and the mixture was kept at 95 °C for 15 min. The radiochemical yield and purity were determined by HPLC and iTLC methods.

#### 2.1.4. Stability Studies of [^99m^Tc]Tc-CIP and [^68^Ga]Ga-DOTA-CIP Radioconjugates

Stability of [^99m^Tc]Tc-CIP and [^68^Ga]Ga-DOTA-CIP previously isolated from the reaction mixture was tested by incubation at 37 °C in 10 mM solutions of strongly competing standard ligands cysteine (CYS) and histidine (HIS) in PBS buffer, pH 7.4. After different time intervals the content of the solutions was tested by HPLC method.

In the case of stability studies in human serum (HS), the solution of isolated radioconjugate in PBS (0.1 mL) was added to 0.9 mL of the human serum and incubated at 37 °C. After time periods up to 4 h, 0.2 mL of the incubated serum mixture were withdrawn, mixed in an Eppendorf tube with 0.5 mL ethanol and vigorously shaken to precipitate proteins. After centrifuging (14,000 rpm for 5 min) the γ-radiation of both the supernatant and precipitate was measured using the well-type NaI(Tl) detector. To check whether the radioconjugate had not converted into other water-soluble radioactive species, aliquots of the supernatant were analysed by HPLC.

#### 2.1.5. Lipophilicity Studies of [^99m^Tc]Tc-CIP and [^68^Ga]Ga-DOTA-CIP Radioconjugates

The lipophilicity of the radioconjugates was characterised by the determination of the logarithm of distribution coefficient, logD, in the n-octanol/PBS (pH 7.40) system, which mimics the physiological conditions (Product Properties Test Guidelines OPPTS 830.7550, 1996). The 1 mL PBS solution of a given radioconjugate previously isolated from reaction mixture was introduced to a test tube containing 1 mL of *n*-octanol and vortexed for 1 min. After that, the test tube was centrifuged for 5 min at 5800 rpm to ensure complete separation of the layers. The activity of each layer was measured, using a well-type NaI(Tl) detector. Distribution coefficient D was calculated as the ratio of activity of the organic to that of the aqueous phase moreover, the aqueous phase was analysed by HPLC to check sample purity and whether the radioconjugate studied did not decompose during the experiment. The logD measurements of all radioconjugates were performed in triplicate. The lipophilicity values determined in our study were compared with the literature relative data without an internal reference.

#### 2.1.6. Electrophoresis Analysis of [^99m^Tc]Tc-CIP and [^68^Ga]Ga-DOTA-CIP Radioconjugates

Paper electrophoresis experiments were achieved on 20 × 1 cm chromatographic paper strips, Paper Chromedia GF 83 (Whatman), in the phosphate buffer (0.01 M, pH 7.40), using the midi horizontal electrophoresis unit (Sigma-Aldrich) device. For both radioconjugates, the experiments were carried out at 200 V (10 V cm^−1^) for 45 min and then the distribution of radioactivity on the strips was determined using a Storage Phosphor System Cyclone Plus (Perkin-Elmer Life and Analytical Sciences, Downers Grove, IL, USA).

#### 2.1.7. [^99^^m^Tc]Tc-CIP and [^68^Ga]Ga-DOTA-CIP Antibacterial Properties Study

The ability of [^99m^Tc]Tc-CIP and [^68^Ga]Ga-DOTA-CIP radioconjugates binding to bacteria was assessed using the Gram-positive bacteria *S. aureus* ATCC 25,923 and Gram-negative *P. aeruginosa* ATCC 27,853 reference strains. Bacteria were cultured on Columbia Agar supplemented with 5% sheep blood (Graso Biotech, Starogard Gdański, Poland) at 37 °C in an aerobic atmosphere for 24 h. After incubation, a bacterial suspension with the turbidity 3 according to the McFarland standards (cell density approximately 9 × 10^8^ CFU/mL) was prepared in PBS. Then, 100 μL of [^99m^Tc]Tc-CIP (3 MBq) or [^68^Ga]Ga-DOTA-CIP (300 kBq) radioconjugate was added to the Eppendorf tube containing 900 μL of the bacterial suspension. The mixtures of bacteria and the radioconjugate were incubated in separated tubes for different time intervals at 37 °C without shaking. After incubation, each test tube was centrifuged at 7000 rpm for 5 min. The supernatant was removed to a new tube, and the bacterial pellet was resuspended in 1 mL of PBS, then the sample was again centrifuged at 7000 rpm for 5 min. After second centrifugation, the supernatant was removed to a new tube and the bacterial pellet was again resuspended in 1 mL of PBS. The radioactivity measurement was performed for all three fractions, two supernatants, and a final bacterial pellet suspension. Experiments for each incubation time were triplicate.

#### 2.1.8. SPECT Imaging with [^99m^Tc]Tc-CIP Radioconjugate

The scintigraphic imaging was performed 4 h after the injection of 700–800 MBq of [^99m^Tc]Tc-CIP, with dual-head, hybrid gamma-camera (Symbia, Siemens) using whole-body and single photon emission tomography/computed tomography (SPECT/CT) techniques. The research was conducted according to the guidelines of the Declaration of Helsinki, and approved by the Bioethics Committee of Medical University of Warsaw (KB/131/2014, 10 June 2014 and KB/62/A/2014, 13 August 2014). Several patients selected for the study had an established diagnosis of DFS and a suspicion of bacterial infection of the foot.

## 3. Results and Discussion

The crude product after DOTA-CIP synthesis was purified using system 1 and lyophilised.

MS for DOTA-CIP; C_33_H_44_FN_7_O_10_, TOF MS ES^+^: Calculated: [M + H]^+^: 718.31 *m*/*z*; Found: [M + H]^+^: 718.34 *m*/*z*

**Figure 3:**
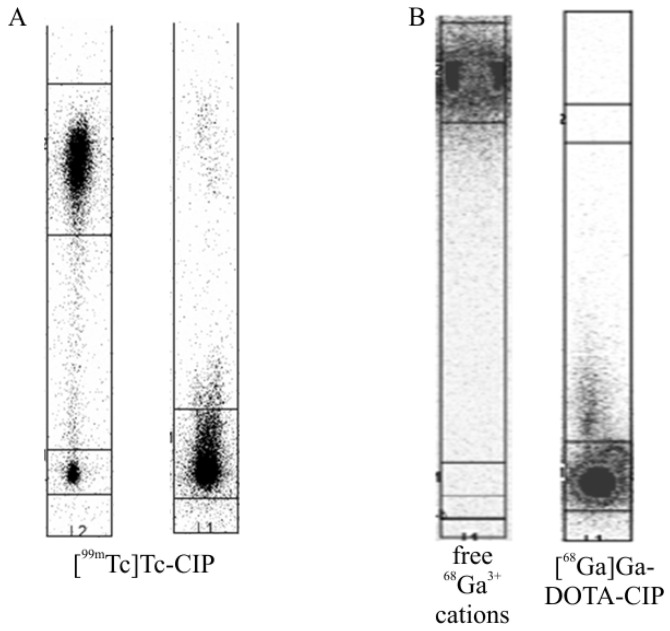
iTLC analyses of reaction mixtures after synthesis of (**A**): [^99m^Tc]Tc-CIP in H_2_O/acetone/NH_3_ (**left**) and in acetone (**right**), and (**B**): [^68^Ga]Ga-DOTA-CIP radioconjugate and free ^68^Ga^3+^ in citrate buffer.

**Figure 4:**
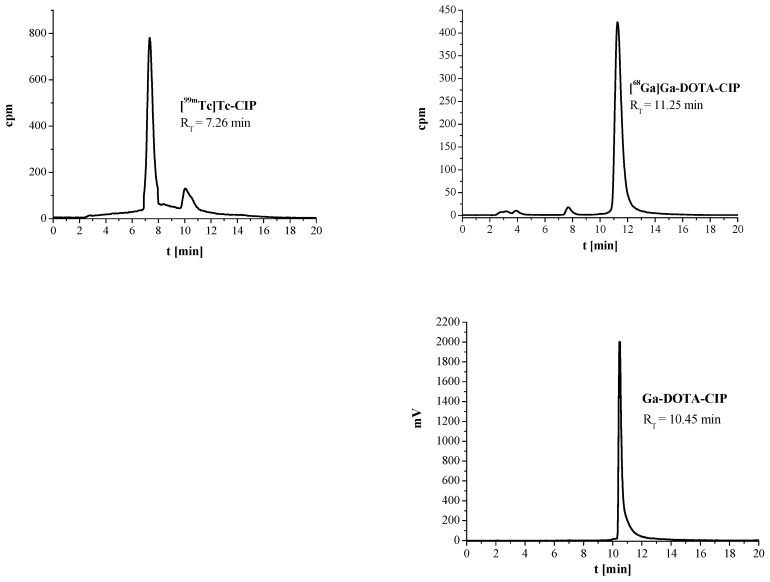
HPLC analyses of reaction mixtures after syntheses of [^99m^Tc]Tc-CIP and [^68^Ga]Ga-DOTA-CIP radioconjugates, and cold reference compound Ga-DOTA-CIP.

Both radioconjugates were synthesised in high yield and radiochemical purity, >90% (Figure 3 and Figure 4). iTLC analysis (Figure 3) of the reaction mixture after the synthesis of [^99m^Tc]Tc-CIP was performed using either a mixture of H_2_O/acetone/NH_3_, 5:1:2 *v/v/v,* or neat acetone as the developing solution. Under these conditions, the [^99m^Tc]Tc-CIP radioconjugate migrated with the solvent front (R_f_ ≈ 1) with the mixed solvent and remained at the origin with neat acetone (R_f_ ≈ 0). In both solvents, the colloidal form of ^99m^Tc remained at the origin, while the pertechnetate anion [^99m^Tc]TcO_4_^−^ migrated with the acetone solvent front. In the case of the [^68^Ga]Ga-DOTA-CIP radioconjugate, 0.2 M citrate buffer, pH 5.0, was used as the developing solution. Under these conditions, free ^68^Ga^3+^ cations (^68^Ge/^68^Ga generator eluate) moved with the solvent front (R_f_ ≈ 1), while the [^68^Ga]Ga-DOTA-CIP radioconjugate remained at the origin (R_f_ ≈ 0.0–0.1).

HPLC analyses of the reaction mixtures after the syntheses of the [^99m^Tc]Tc-CIP and [^68^Ga]Ga-DOTA-CIP radioconjugates (system 2) and also a cold reference compound Ga-DOTA-CIP (system 1) are presented in Figure 4.

The HPLC chromatogram of the cold Ga-DOTA-CIP reference compound (Figure 4, system 1, the preparation of this compound was confirmed by MS analysis) showed a single peak with R_T_ = 10.45 min, corresponding to the R_T_ value of [^68^Ga]Ga-DOTA-CIP radioconjugate (R_T_ = 11.25 min). The slight difference between the positions of the peaks resulted from the arrangement in series of the gamma detector after the UV/Vis detector. The peak position of the cold reference compound confirmed that the corresponding radiopreparation was obtained in the labelling reactions.

Ga-DOTA-CIP MS for C_33_H_42_FGaN_7_O_10_, TOF MS ES^+^ Calculated: [M + H]^+^: 784.22 *m*/*z*; Found: [M + H]^+^: 784.31 *m*/*z*
CIP ^1^H-NMR: (D_2_O, 500 MHz, in ppm, T = 295K): 1.17, 1.41 (both 2H, –CH_2_– cyclopropyl), 3.51, 3.61 (both 4H, –CH_2_– piperazynyl), 3.66 (1H, CH, cyclopropyl), 7.33 (dd, 1H, H-5), 7.40 (d, 1H, H-8), 8.53 (s, 1H, H-2)
DOTA-CIP ^1^H-NMR: (D_2_O, 500 MHz, in ppm, T = 295K): 1.20, 1.42 (–CH_2_– cyclopropyl), 2.70, 2.84 (–CH_2_–COOH), 3.28–3.85 (N–CH2–CH2–N, –CH_2_– piperazinyl, CH cyclopropyl), 7.60 (H-5), 7.77 (H-8), 8.81 (H-2)
Ga-DOTA-CIP ^1^H-NMR: D_2_O, 500 MHz, in ppm, T = 295K): 1.22, 1.40 (–CH_2_– cyclopropyl),1.98, 2.78 (–CH_2_–COOH), 3.25–4.21 (N–CH_2_–CH_2_–N, –CH_2_– piperazinyl, CH cyclopropyl), 7.49 (H-5), 7.77 (H-8)

The observed changes in chemical shifts of CIP after the reaction in DOTA indicate the formation of DOTA-CIP complex. Similarly, further changes can be observed in the ^1^H NMR spectrum registered for Ga-DOTA-CIP, including the observation of multiple resonances corresponding to –N–CH_2_–CH_2_–N moieties. This suggests increased rigidity of this fragment of a molecule upon complexation of Ga.

The results of physicochemical studies (R_T_ values, stability in CYS, HIS, and HS, and the lipophilicity parameter) for the [^99m^Tc]Tc-CIP and [^68^Ga]Ga-DOTA-CIP radioconjugates are presented in Table 1.

**Figure 5:**
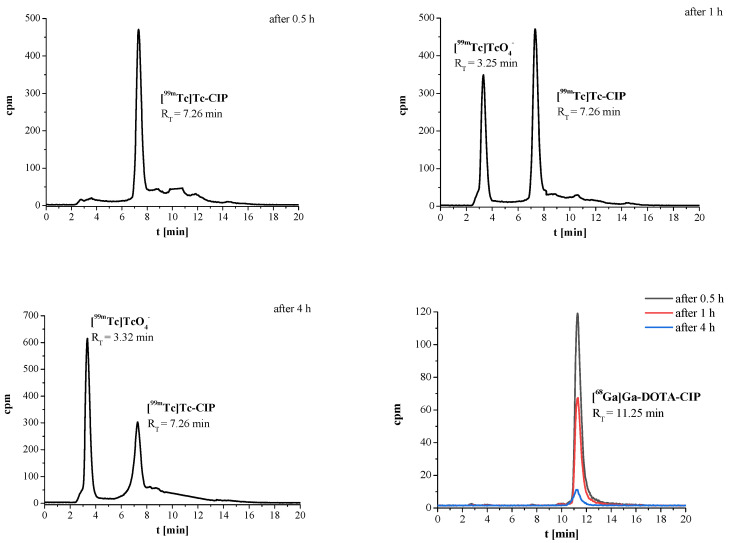
HPLC radiochromatograms of stability of [^99m^Tc]Tc-CIP and [^68^Ga]Ga-DOTA-CIP radioconjugates during incubation in HS.

The results of stability studies for the [^99m^Tc]Tc-CIP and [^68^Ga]Ga-DOTA-CIP radioconjugates in HS are also presented in Figure 5. As can be seen (Table 1), the radiocompound [^99m^Tc]Tc-CIP in either CYS or HIS challenging solutions was stable over a time corresponding to the period of four half-lives of the Tc-99m radionuclide; however, in HS it slowly decomposed. On the HPLC radiochromatograms, two peaks were visible, with R_T_ values of 3.32 min (recognised as [^99m^Tc]TcO_4_^−^) and 7.26 min ([^99m^Tc]Tc-CIP). Simultaneously with the disappearance of the [^99m^Tc]Tc-CIP peak, the [^99m^Tc]TcO_4_^−^ peak increased, and after an incubation time of 4 h (less than the half-life of the Tc-99m radionuclide), only about 45% of the [^99m^Tc]Tc-CIP radioconjugate remained intact. In contrast, the [^68^Ga]Ga-DOTA-CIP radioconjugate was completely stable in CYS, HIS, or HS solutions during a period corresponding to four half-lives of the Ga-68 radionuclide. HPLC radiochromatograms were recorded with only single peaks with an R_T_ value corresponding to that of the [^68^Ga]Ga-DOTA-CIP radioconjugate (the decreasing peak height was related to the radionuclide decay).

**Figure 6:**
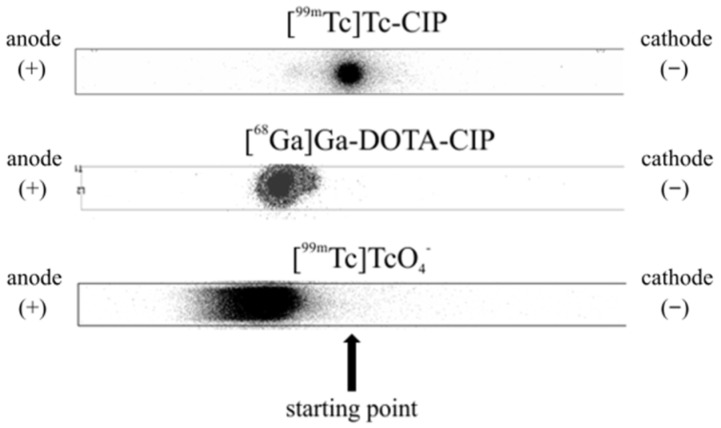
Electrophoresis analyses of [^99m^Tc]Tc-CIP, [^68^Ga]Ga-DOTA-CIP and [^99m^Tc]TcO_4_^−^ radiocompounds in 0.01 M phosphate buffer pH 7.40.

Electrophoresis analyses of the [^99m^Tc]Tc-CIP and [^68^Ga]Ga-DOTA-CIP radioconjugates are presented in Figure 6. The same figure also shows the electrophoresis analysis of the well-defined pertechnetate anion [^99m^Tc]TcO_4_^−^.

Based on the results presented, the [^99m^Tc]Tc-CIP radioconjugate can be considered to be an uncharged molecule. As the molecular structure of this radiocompound is still unknown, it is difficult to discuss the oxidation state of the Tc-99m cation and the distribution of point charges in the molecule. Electrophoresis analysis of the [^68^Ga]Ga-DOTA-CIP radioconjugate showed that this molecule has a negative charge, most likely resulting from the dissociation of the carboxyl group of the DOTA chelator, which does not participate in the formation of a complex with the ^68^Ga^3+^ cation.

Experimental results for a binding study of [^99m^Tc]Tc-CIP and [^68^Ga]Ga-DOTA-CIP radioconjugates with *S. aureus* and *P. aeruginosa* are presented in Table 2. The bacterial uptake of [^99m^Tc]Tc-CIP was significantly higher than that of [^68^Ga]Ga-DOTA-CIP in the case of both bacterial species. Moreover, the uptake of the [^99m^Tc]Tc-CIP radioconjugate by *S. aureus* was visibly higher than that by *P. aeruginosa*, which may suggest that CIP is a better vector for Gram-positive bacteria (e.g., *S. aureus*—the most common bacterium in DFS infection). In the case of [^68^Ga]Ga-DOTA-CIP, this difference was insignificant (within the error limits), and the uptake of the radiopreparation by the bacteria was comparable for both species.

**Figure 7:**
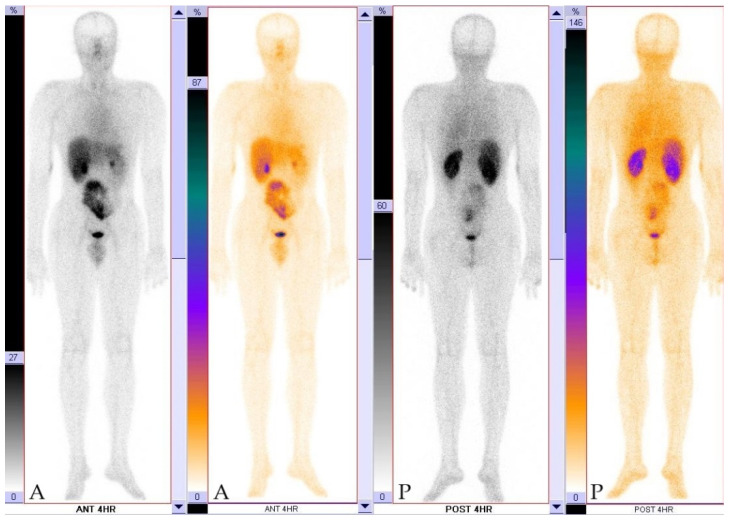
Whole-body [^99m^Tc]Tc-CIP scintigraphy of a healthy patient 4 h (A-anterior and P-posterior) after administration of the radiotracer. Visible physiological radiotracer accumulation mainly in the liver and kidneys.

**Figure 8:**
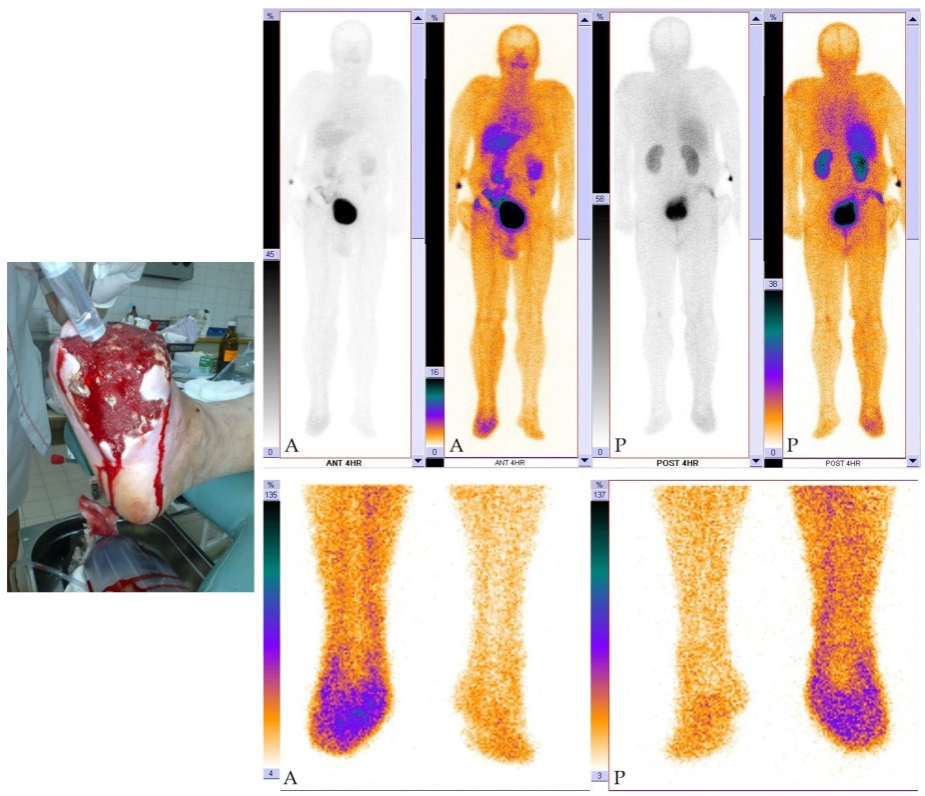
Morphological image of the foot infected in course of the DFS and whole-body [^99m^Tc]Tc-CIP scintigraphy of a patient with infected diabetic right foot (A-anterior and P-posterior) in course of the DFS. Intense radiotracer uptake in the bladder, liver, kidneys, as well in the infected part of the right foot.

**Figure 9:**
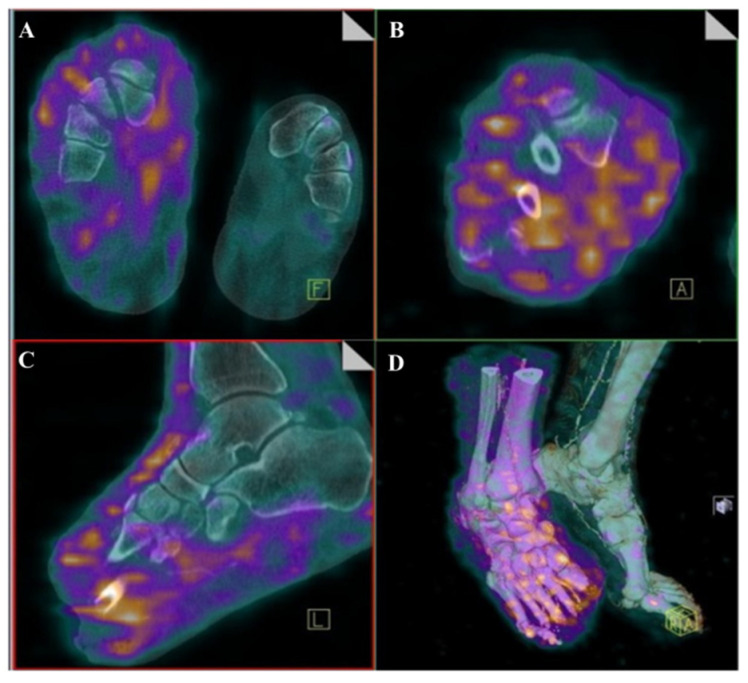
SPECT/CT of the feet of diabetic with a very advanced DFS. (**A**) Transverse cross-section of both feet. (**B**) Coronal cross-section of the right foot. (**C**) Sagittal cross-section of the right foot. (**D**) Three-dimensional reconstruction of both feet with visualization of bone tissues.

[^99m^Tc]Tc-CIP was found to be a neutrally charged and slightly hydrophilic compound with a relatively low molecular weight, which most likely promoted its binding to bacterial cells. A relatively significant disadvantage of this preparation was its unsatisfactory stability in the serum; nevertheless, SPECT imaging of the DFS gave very good results (Figure 7, Figure 8 and Figure 9). Further disadvantages of this preparation were its unknown structure and the fact that its synthesis in the labelling reaction required a relatively large amount of antibiotics—of the order of 2 mg per kit. The second radioconjugate, [^68^Ga]Ga-DOTA-CIP, had a comparable molecular weight, a well-defined structure and showed complete stability in HS. However, it was negatively charged and was characterised by an almost two times lower lipophilicity parameter, which could be the reason for its poor binding ability to bacterial cells. We also compared the binding to *S. aureus* cells of [^68^Ga]Ga-DOTA-CIP with the data for the [^68^Ga]Ga-DOTA-Bz-SCN-PA-CIP and [^68^Ga]Ga-NOTA-Bz-SCN-PA-CIP radiopreparations synthesised and tested (under the same experimental conditions) by Satpati et al. [21]. The binding uptake of these radiotracers by bacterial cells was approximately 1% and 2%, respectively, and the results obtained for [^68^Ga]Ga-DOTA-Bz-SCN-PA-CIP were very similar to the value determined by us for [^68^Ga]Ga-DOTA-CIP (Table 2). In the analysis of the dependence between radiopreparation lipophilicity and their binding to bacteria, we also used theoretically calculated (using the ALOGPS 2.1 and ChemOffice programs) the lipophilicity values of the [^68^Ga]Ga-DOTA-CIP, [^68^Ga]Ga-DOTA-Bz-SCN-PA-CIP and [^68^Ga]Ga-NOTA-Bz-SCN-PA-CIP radioconjugates (Table 3). As can be seen, the numerical data obtained by the use of different calculation programs were radically different and certainly cannot be strictly accepted as values for the lipophilicity parameters of the studied radiocompounds. Nevertheless, these values, calculated for all three radioconjugates with the same assumptions of the calculating program, allow the trend in the lipophilicity parameter of these radiopreparations to be estimated [24]. Comparing the structures of [^68^Ga]Ga-DOTA-CIP, [^68^Ga]Ga-DOTA-Bz-SCN-PA-CIP, and [^68^Ga]Ga-NOTA-Bz-SCN-PA-CIP, and based on our own experience, we believe that the lipophilicity of these three radiocompounds is represented by the arrangement presented in Table 3.

Based on the data presented in Table 2 and Table 3, it can be concluded that the more lipophilic CIP-based radioconjugates are bound more efficiently by bacterial cells.

The use of [^99m^Tc]Tc-CIP radiotracer for infection imaging in DFS is illustrated in Figure 7, Figure 8 and Figure 9. Figure 7 shows whole body imaging of a healthy person 4 h after administration of the radiotracer. Physiological uptake was visible in the liver, spleen, kidneys, and bladder, as well as being slightly visible in the salivary glands and intestines. The lack of visible Tc-99m radionuclide accumulation in the thyroid gland suggested a lack of unbound/non-reduced Tc-99m forms.

Figure 8 and Figure 9 show a morphological image of the foot of a diabetic with very advanced DFS and [^99m^Tc]Tc-CIP scintigraphy of the same patient. Whole-body scintigraphy (Figure 8) showed high radiotracer accumulation in the bladder, liver, and kidneys, as well as in the infected part of the right foot.

Figure 9 presents SPECT/CT imaging of the patient’s feet. Intense radiotracer uptake was visible in the right foot and a lack of uptake in the left foot (Figure 9A,D). Imaging of the infected right foot showed high radiotracer uptake in soft tissues (Figure 9A,C), no uptake in the tarsal bones (Figure 9C), partial amputation of the toes (Figure 9C,D), and severe soft tissue oedema in the forefoot and midfoot (Figure 9A–C). The lack of accumulation of [^99m^Tc]Tc-CIP radiotracer in the bone tissue possibly suggested no bone infection, which may have been an indication for further antibiotic therapy and no amputation.

## 4. Conclusions

Based on the physicochemical and biological properties of CIP-based radiopreparations, it can be considered that CIP is a good vector for delivering radiopreparations to the sites of bacterial infection. When assessing the potential use of [^99m^Tc]Tc-CIP and [^68^Ga]Ga-DOTA-CIP radiotracers, it can be concluded that both radiopreparations can be used for DFS imaging by SPECT or PET, respectively. Although the [^68^Ga]Ga-DOTA-CIP radiopreparation was characterised by a lower bacterial uptake (depending on the lipophilicity parameter of the radiopreparation), PET has a higher sensitivity as well as higher spatial and temporal resolution, along with the possibility of quantification, which allows for precise imaging.

## Figures and Tables

**Table 1:** Results of physicochemical studies of [^99m^Tc]Tc-CIP and ^68^Ga]Ga-DOTA-CIP radioconjugates.

Radioconjugate			[^99m^Tc]Tc-CIP		[^68^Ga]Ga- DOTA-CIP	
R_T_			7.26 min		11.25 min	
% of intact radioconjugate after incubation in:	CYS		after 24 h	~100	after 4 h	~100
	HIS			~100		~100
	HS after	0.5 h	98		~100	
		1 h	65		~100	
		4 h	45		~100	
logD			−1.8 ± 0.1		−3.1 ± 0.1	

**Table 2:** Results of the in vitro [^99m^Tc]Tc-CIP and [^68^Ga]Ga-DOTA-CIP binding study to *Staphylococcus aureus* and *Pseudomonas* *aeruginosa*.

Species	Binding Time [min]	Radioconjugate Uptake [%]	
		[^99m^Tc]Tc-CIP	[^68^Ga]Ga-DOTA-CIP
*Staphylococcus aureus*	30	8.77 ± 0.4	0.89 ± 0.11
	60	12.75 ± 1.02	1.1 ± 0.15
*Pseudomonas aeruginosa*	30	5.02 ± 0.43	0.82 ± 0.16
	60	8.89 ± 0.61	1.29 ± 0.27

**Table 3:** Evaluation of lipophilicity parameter of CIP-based radioconjugates containing ^68^Ga radionuclide complex.

Radioconjugate	[^68^Ga]Ga-DOTA-CIP, (1)	[^68^Ga]Ga-DOTA- Bz-SCN-PA-CIP, (2)	[^68^Ga]Ga-NOTA- Bz-SCN-PA-CIP, (3)
Elements of the radioconjugate molecule structure	-----	Lipophilic fragment Bz-SCN-PA	Lipophilic fragment Bz-SCN-PA
	Free carboxyl group of the DOTA chelator	Free two carboxyl groups of the DOTA chelator	-----
	Negative charge	Negative charge *^1^	Neutral molecule *^2^
Lipophilicity	−3.1 ± 0.1	No data *^3^	No data *^4^
Theoretical lipophilicity values *^5^	−0.95	−0.84	−0.25
Theoretical lipophilicity values *^6^	−5.51	−5.97	−4.20
	logD_(1)_ ≈ logD_(2)_ < logD_(3)_.		

*^1^ in ref. [21] there is no data of the electrophoresis study of radiocompound (2), however in the molecule there are two free carboxyl groups of the DOTA chelator; *^2^ in ref. [21] there is no data of electrophoresis study of radiocompound (3), however in the molecule there is no free carboxyl group; *^3^ in ref. [21] there is no data of lipophilicity study of radiocompound (2), however in the molecule there is the additional lipophilic fragment Bz-SCN-PA; *^4^ in ref. [21] there is no data of lipophilicity study of radiocompound (3), however in the molecule there is the additional lipophilic fragment Bz-SCN-PA and there is no free carboxyl group. *^5^ theoretically calculated lipophilicity values using ALOGPS 2.1 program. *^6^ theoretically calculated lipophilicity values using the ChemOffice program.

## Data Availability

Not Applicable.
